# Soft Tissue Special Issue: Perivascular and Vascular Tumors of the Head and Neck

**DOI:** 10.1007/s12105-020-01129-z

**Published:** 2020-01-16

**Authors:** Uta Flucke, Marie Karanian, Roel W. ten Broek, Khin Thway

**Affiliations:** 1 Department of Pathology, Radboud University Nijmegen Medical Center, P.O. Box 9101, 6500 HB Nijmegen, The Netherlands; 2 Princess Maxima Center for Pediatric Oncology, Utrecht, The Netherlands; 3 Department of Pathology, Léon Bérard Center, University Claude Bernard Lyon, Lyon, France; 4 Sarcoma Unit, Royal Marsden Hospital, London, UK

**Keywords:** Perivascular neoplasms, Hemangioma, Vascular malformations, Angiosarcoma, Hemangioendothelioma

## Abstract

Perivascular and vascular neoplasms of the head and neck are a rare group of tumors comprising a spectrum of clinical/biologic and histological features. They are frequently diagnostically challenging, due to their morphologic and immunohistochemical overlap. In this review, we summarize the pathology of these neoplasms, discussing morphology, immunohistochemistry, associated genetic findings, and the differential diagnoses.

## Perivascular Tumors

Perivascular tumors in the head and neck region are benign, and comprise myofibroma/myopericytoma, angioleiomyoma, nasopharyngeal angiofibroma, sinonasal glomangiopericytoma/sinonasal-type hemangiopericytoma and more rarely glomus tumor.

## Myofibroma

**Fig. 1:**
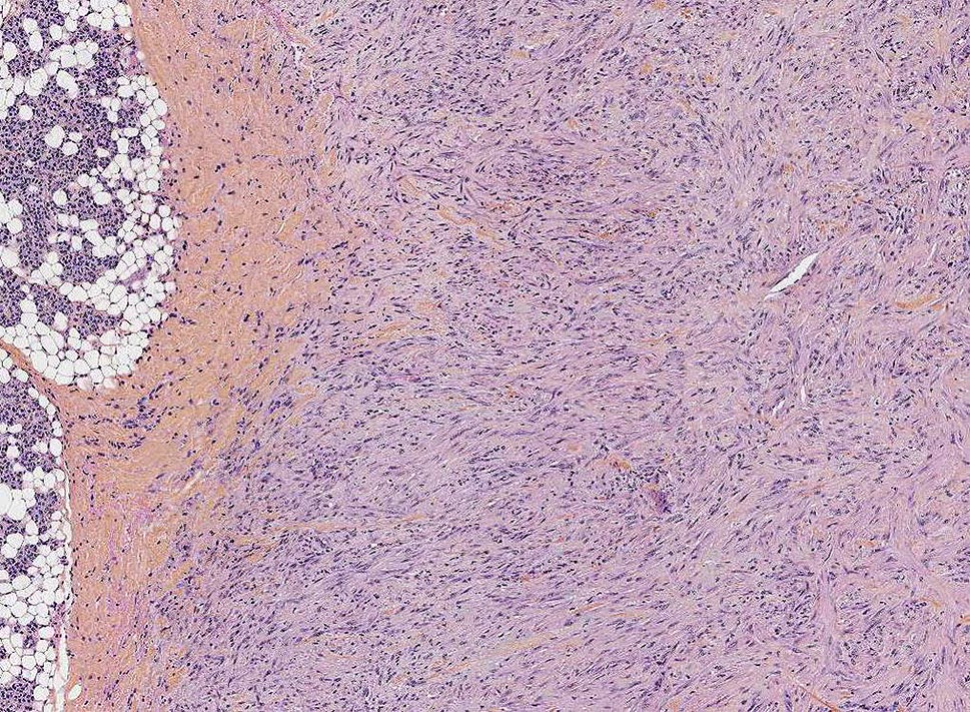
Myofibroma with infiltration of the parotid gland. Myofibroblastic cells are embedded in a fibromyxoid stroma. This lesion showed a *PDGFRB* mutation

This is a benign myofibroblastic lesion, that forms a morphological continuum with myopericytoma and angioleiomyoma. Lesions occur at any age, although children are more often affected and more frequently present with multifocal disease. Myofibromas of the head and neck arise commonly as small nodules located submucosally or subcutaneously. The oral cavity, including tongue and gingiva are most often involved. Tumors may also affect the dermis and bone as primary or secondary sites. Macroscopically, the nodules are sharply or ill-defined with a firm grey-white appearance. Microscopically, ill-defined neoplasms show infiltrative growth. There is generally a biphasic pattern in varying proportions consisting of a spindle cell component with tapered nuclei set in a myxohyaline matrix and cellular areas with plump spindle or round cells surrounding a branching hemangiopericytoma-like vasculature. Cellular atypia is absent, but mitotic figures may be observed. Intravascular extension is a typical feature within the spindle cell myxohyaline areas (Fig. 1). Immunohistochemically, smooth muscle actin (SMA) is positive with variable expression, while desmin is expressed in a subset of cases [1–4]. Genetically, recurrent somatic *PDGFRB* mutations are reported in the majority of cases [5]. In cellular forms of myofibromas/myopericytomas a *SRF*-*RELA* fusion gene has been described [6].

Although the differential diagnosis includes other myofibroblastic lesions (e.g. desmoid-type fibromatosis, nodular fasciitis, infantile fibrosarcoma) and deep benign fibrous histiocytoma/cellular dermatofibroma, the typical biphasic pattern and the myxohyaline matrix of myofibromas is not observed in these lesions and genetic changes are different (desmoid: beta-catenin mutations; nodular fasciitis: *USP6*-rearrangements, infantile fibrosarcoma: *ETV6*-*NTRK3* fusion) [4].

## Myopericytoma

This lesion shows overlapping morphologic features of myofibroma and angioleiomyoma with typically circumscribed, lobular, variably cellular distributions of bland, ovoid to spindled myoid cells growing around mainly small vessels in a concentric, multilayered fashion [1, 3, 7].

## Angioleiomyoma

Angioleiomyoma is a relatively uncommon benign lesion consisting of prominent mainly thick-walled vessels intermingled with surrounding smooth muscle cells. It forms a morphological spectrum with myofibroma and myopericytoma and arises usually in skin and subcutis. Occurrence in the head and neck is rare with an incidence of up to 13% with variable sites, e.g. intraoral, salivary glands and sinonasal. Adults are mainly affected. Lesions can be painless, tender or painful. Grossly, they are polypoid, nodular and sharply demarcated with a whitish trabecular cut surface with sponge-like areas with blood. Histologically, well-differentiated smooth muscle cells are intimately associated with a prominent thick-walled vascular component with a concentric layer of smooth muscle. The lumina can be collapsed or dilated. Mitotic figures are usually absent. Nuclear atypia, myxoid changes, calcification, ossification and fatty metaplasia can be present in longstanding regressive lesions [7–10]. One peculiar angioleiomyoma in the lymph node hilus usually occurs in the neck [10]. Immunohistochemical expression of smooth muscle markers as SMA, desmin and caldesmon is typically observed. Simple excision is the adequate therapy [7–10]. Myopericytoma could be a differential diagnosis but belongs to the same spectrum. EBV-associated smooth muscle tumors may, as common smooth muscle tumors, mimic angioleiomyoma but these rare lesions are characteristically multifocal and affect patients with immunodeficiency. A prominent vasculature is not expected. Ebstein-Barr Virus is detectable by in situ hybridization. Lesions with adipocytic metaplasia could be confused with the very rare angiomyolipomas belonging to the PEComa group. Melanocytic markers as HMB45 and Melan A can help to make a distinction [9].

## Nasopharyngeal Angiofibroma (JNA)

(Juvenile) nasopharyngeal angiofibroma (JNA) is an uncommon benign fibrovascular lesion originating in the nasopharyngeal region of mainly male adolescents and young adults aged between 14 and 25 years. Women and older patients are rarely affected [11–13]. JNA involves the nasopharynx and dorsolateral aspect of the nasal cavity. Although morphologically benign it can cause destructive involvement of the paranasal sinuses, orbit and skull base with intracranial extension [12, 13]. Symptoms are commonly obstruction and epistaxis. Individual cases have been associated with familial adenomatous polyposis [12, 14]. Recurrences after surgery are reported in up to 57% of cases [11].

**Fig. 2:**
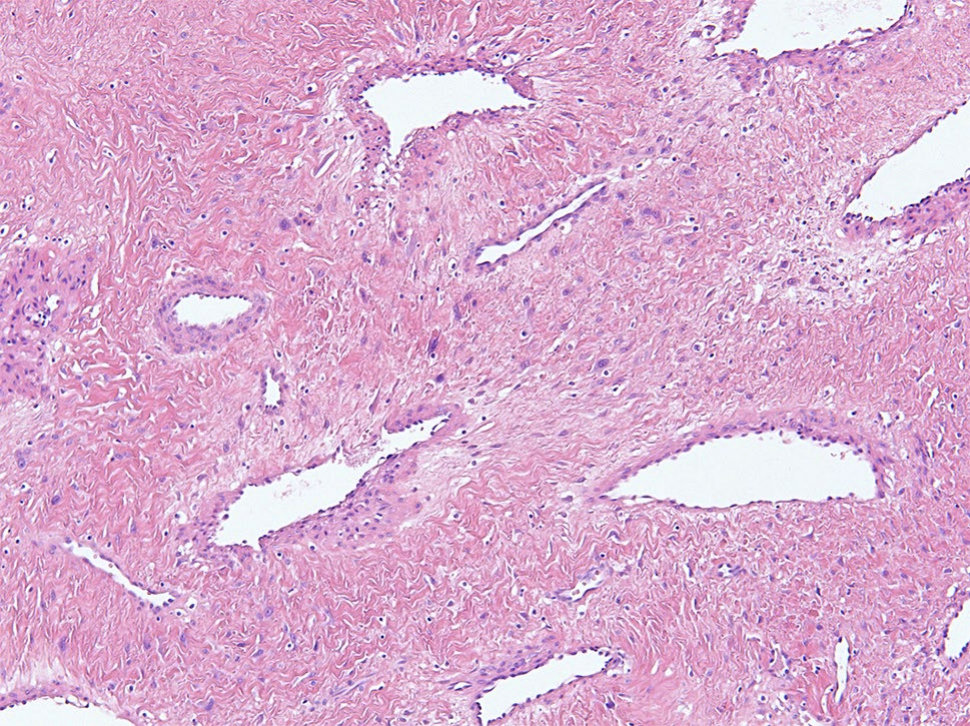
Nasoparyngeal angiofibroma is characterized by a disorganized prominent vasculature with an inconsistent smooth muscle layer and a fibrous stroma

These polypoid submucosal smooth lesions consist histologically of disorganized large vessels with an inconsistent smooth muscle layer, which is a hallmark. The surrounding fibrous stroma is variably cellular and shows fibroblasts with oval and tapered nuclei and inconsistently distinct nucleoli (Fig. 2). Small numbers of mitotic figures can be present. Multinuclear cells and pleomorphism may be seen being a sign of regression. Immunohistochemically, nasopharyngeal angiofibromas are SMA positive, highlighting the incomplete vascular muscle layer. Nuclear expression of beta-catenin is a result of mutations in the corresponding *CTNNB* or *ABC* gene, leading to reduced degradation of the protein. Androgen receptor expression is also typical for this lesion [11, 12, 14]. Benign vascular lesions as vascular malformations could be a differential diagnosis. However they have a different genetic background (see below).

## Sinonasal Glomangiopericytoma/Sinonasal-Type Hemangiopericytoma (s-HPC)

These mesenchymal lesions of low-biologic potential occur exclusively in the nasal cavity and paranasal sinuses. Middle-aged adults of either sex are mainly affected. Patients typically present with nasal obstruction and epistaxis [2, 15, 16]. Surgery is the treatment of choice, with recurrences reported in a small number of patients [15].

**Fig. 3:**
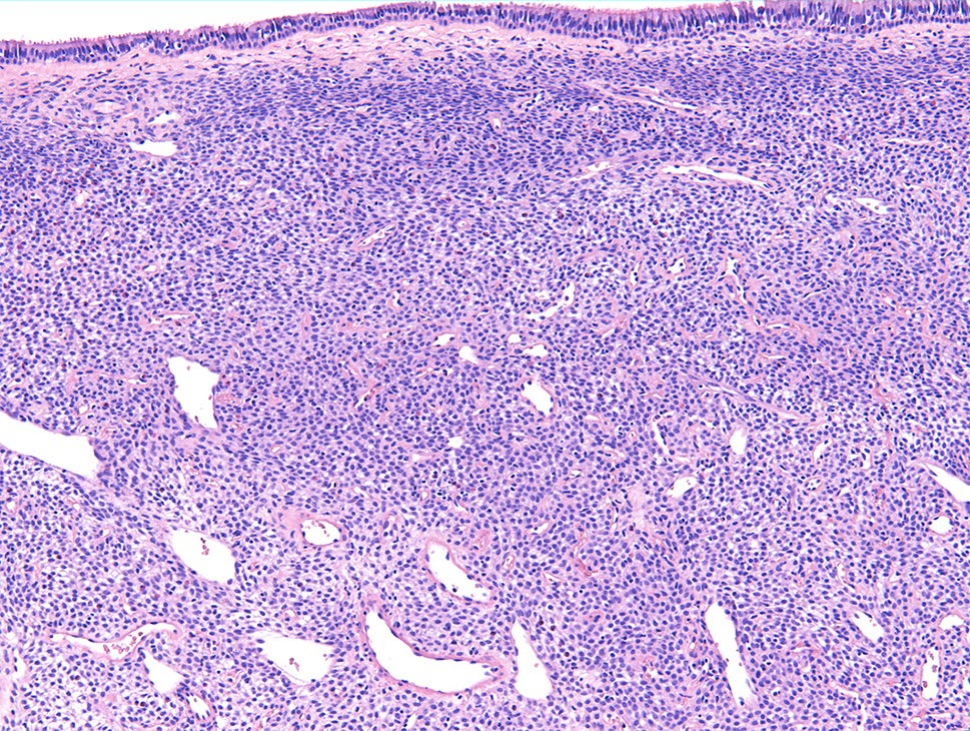
Sinonasal hemangiopericytoma consists of monomorphic spindle cells with oval nuclei. Note the subepithelial grenz zone and the staghorn-like vasculature

Grossly, these tumors are polypoid and nondestructive, with a firm cut surface. Microscopy shows a submucosal nodular, unencapsulated neoplasm with a “Grenz zone” underneath the epithelial surface consisting of monomorphic glomus- or pericytic-like cells with oval to elongated nuclei with smooth contours. The cells are arranged in sheets and fascicles around a prominent vasculature often with a staghorn-like configuration and hyalinization (Fig. 3). The immunohistochemical key marker is beta-catenin, which shows nuclear expression due to an activating hot spot mutation in *CTNNB1.* CD34, SMA and panactin are variably expressed while desmin, S100, keratins and STAT6 are consistently negative [16–18].

A variety of neoplasms enter the morphological differential diagnoses: solitary fibrous tumor can be discriminated by its random or patternless architecture, more tapered nuclei and a variable collagenous background. Nuclear expression of STAT6 due to a *NAB2*-*STAT6* fusion gene is a consistent finding of SFT. Monophasic synovial sarcoma typically shows higher cellularity, with cells disposed in long fascicles. A staghorn-like vasculature may also be present. Synovial sarcoma focally expresses a range of keratins and/or epithelial membrane antigen (EMA) and harbor a specific fusion gene (*SS18*-*SSX1/2*). Malignant peripheral nerve sheath tumor (MPNST) has a variety of morphological appearances, but many are typically also arranged in long fascicles, classically with perivascular cuffs. The cells of MPNST typically show at least focal variable pleomorphism and atypia. Focal positivity for SOX10, S100 and GFAP may help in the diagnosis [18]. Loss of H3K27me3 is substantial in high-grade MPNST, and is therefore useful in the diagnostic work-up [19]. Low-grade biphenotypic sinonasal sarcoma shows rather elongated overlapping nuclei, and neural (S100) and myogenic (SMA, desmin, myogenin, myoD1) immunohistochemical features. Nuclear positivity for PAX3 reflects the presence of a fusion gene involving typically *PAX3 [*20, 21].

## Glomus Tumor

Glomus tumors are rare lesions that occur only exceptionally in the head and neck region, and should not to be confused with paraganglioma, which is often located in the carotid, vagus or middle ear region, for which the term glomus tumor was previously used. The age distribution of glomus tumor is broad, although adults are mainly affected. Presenting symptoms depend on the anatomic site, which includes the oral cavity, larynx and neck [22–24].

Most glomus tumors behave in a benign fashion, and simple excision is the treatment of choice, with only rare recurrences described [23].

Histologically, these nodular lesions typically comprise uniform glomus cells arranged in sheets surrounding small capillaries. The glomus cells are rounded with a centrally placed nucleus, abundant amphophilic cytoplasm and distinct cell borders. Symplastic glomus tumors show marked atypia without mitotic activity (ancient features), while very rare malignant cases additionally show mitotic figures. SMA is strongly expressed, with accentuation of cell borders [24, 25]. Approximately 50% of glomus tumors harbor a *MIR143*-*NOTCH* gen fusion [22].

Glomus tumors are quite distinctive. Epithelial lesions can be excluded by keratin immunohistochemistry.

## Benign Vascular Lesions (Vascular Anomalies)

Benign vascular lesions are divided into hemangioma and vascular malformations. They comprise a diverse group of distinct clinicopathologic entities [26–28].

Distinction between angiomas and vascular malformations as defined by the ISSVA (International Society for the Study of Vascular Anomalies) is encouraged to enable better understanding of lesions etiology, biology and clinical behavior with the aim of improving therapy and patient outcome [29].

Vascular tumors arise by clonal cellular proliferation of vessels showing a disproportionate growth. In contrast, vascular malformations develop in utero as a result of mosaic mutations leading to erroneous development of (mostly heterogeneous) vessels with proportionate growth [27, 28].

The term (capillary) hemangioma does not function well as a stand-alone diagnosis. Instead, pathologists and clinicians should modify this interpretation, indicating specific entities (e.g. infantile hemangioma, non-involuting congenital hemangioma, epithelioid hemangioma, tufted hemangioma) [28].

## Infantile Hemangioma (IH) (Juvenile Hemangioma, Cellular Hemangioma)

These are the most common tumor of infancy, affecting approximately 4% of children with most lesions arising in the skin of the head and neck [27, 28, 30]. Females are more commonly affected. Lesions are not present at birth, but a small purple area is often a precursor lesion [28, 30] Lesions become apparent at 3 to 7 weeks of age proliferating for an average of 3 to 5 months. Involution then occurs over several years, with a variable fibrofatty residuum. IH can arise superficially and/or deeply. Whereas superficial lesions are elevated, those that are deep seated form a mass. There are three distinct morphological patterns: solitary, segmental/diffuse, or multifocal with a considerable variation in size. A biopsy is rarely required due to a clear clinical diagnosis in most cases [28, 30]. Although all IH involute spontaneously over a period of years, significant cosmetic and, in some cases, functional sequelae may occur as ulceration, bleeding, infection, airway obstruction, amblyopia, disfigurement and rarely congestive heart failure [28, 31]. Treatment options are propranolol (first-line), and less common corticosteroids (intralesional or systemic) or surgery [26, 30, 31].

**Fig. 4:**
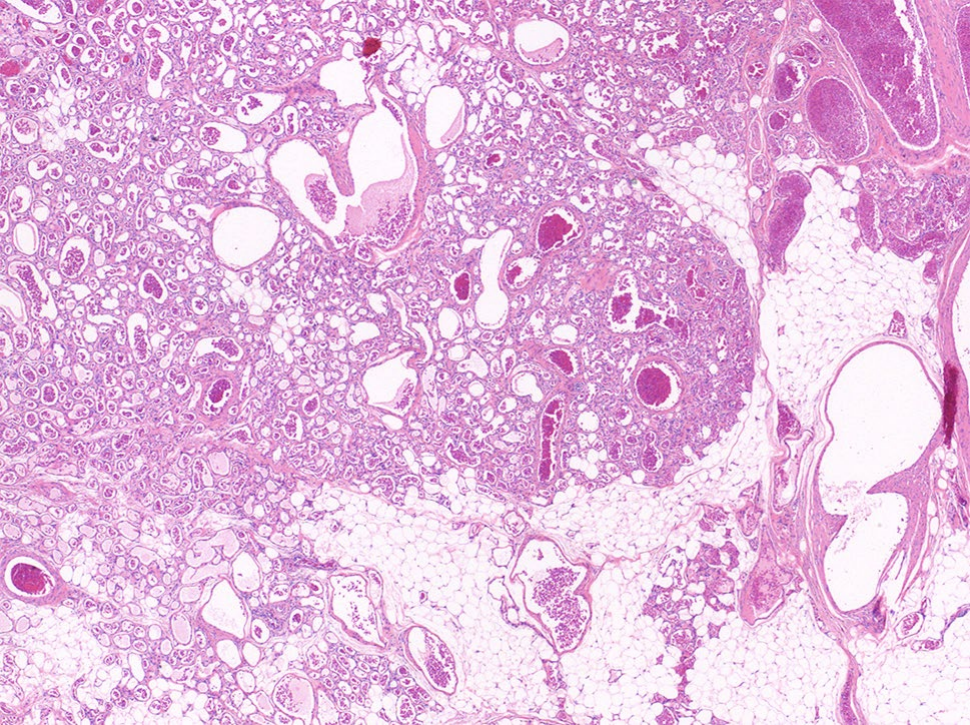
Infantile hemangiomas are characterized by a lobulated architecture. Note the maturated capillaries

**Fig. 5:**
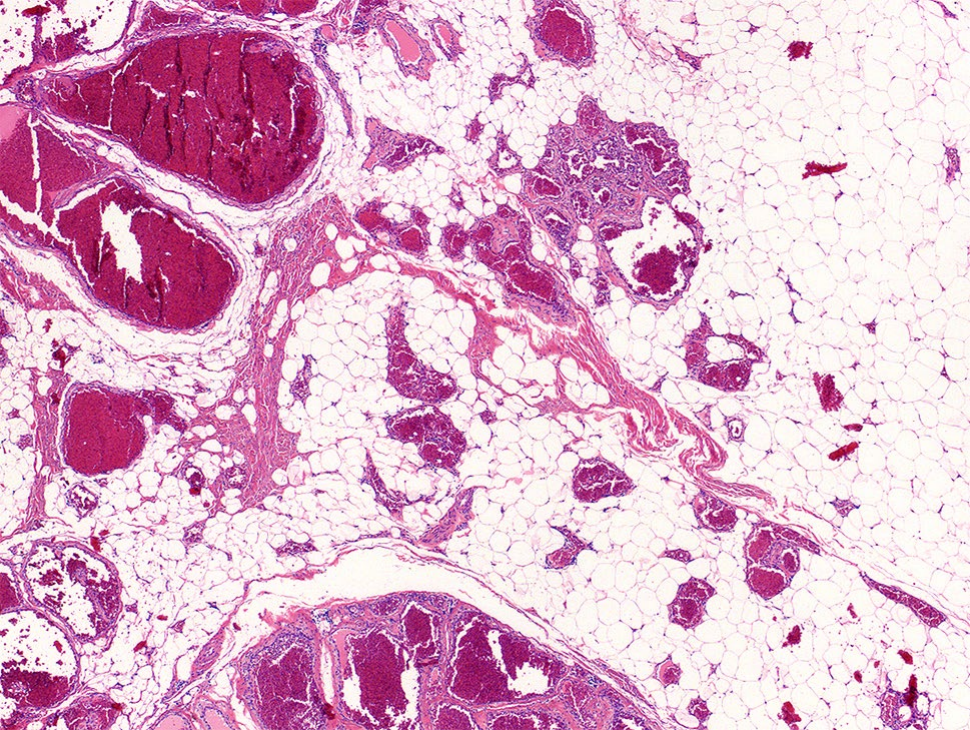
Infantile hemangioma with signs of regression and remaining dilated veins resembling venous malformation

Although histologically these lesions all have a lobular appearance, the morphology can otherwise vary with primitive-looking small, sometimes very subtle lumina and epithelioid endothelial cells in the early stage. In mid-proliferation, capillary formation is more pronounced, with still plump endothelium (Fig. 4). In the late, involutive stage, capillaries disappear to be replaced by scar-like tissue, and endothelial cells are flattened. Involuted lesions can look like vascular malformations because of the residual arteries and veins of abnormal caliber (Fig. 5). However, the nondestructive coexistence of capillaries with peripheral nerves, adipocytes, superficial skeletal muscles and salivary gland is typical and is not observed in other benign vascular lesions. Anastomosing vascular channels, abnormal mitoses and spindled cells are not a feature of IH. Additionally, intravascular thrombosis, hemosiderin deposition and necrosis are absent unless there is extensive ulceration or embolization [28]. Immunohistochemically, various vascular markers highlight the endothelial component, while SMA accentuates the consistent pericytic layer. GLUT1 expression discriminates IH from other benign vascular lesions [28, 31].

## Congenital Hemangioma (CH): Rapidly Involuting (RICH), Partially Involuting (PICH), or Non-involuting (NICH)

Congenital hemangiomas are rare. Although they resemble classic infantile hemangioma, they are now recognized as a pathogenetically distinct lesion. CH manifests in utero/at birth and can affect the head and neck region. They can regress rapidly (more rapidly than IH) usually within a year, with a lipatrophic end-stage or they persist (NICH). Their histomorphology is similar to IH. However there are few mitotic figures, and a sclerotic stroma surrounding the lobules. The intraneural infiltration seen in IH is not a feature of CH and is a useful discriminating morphological feature. Additionally GLUT1 is negative [26, 31]. Somatic activating mutations in GNAQ and GNA11 were consistently found [32]. Treatment is only required in case of persistence, for cosmetic reasons [31].

## Pyogenic Granuloma (Lobular Capillary Hemangioma)

This is a common, acquired vascular tumor with an estimated prevalence of 0.5–1% mainly affecting middle-aged adults. It often occurs in the head and neck, most commonly in the lip and oral cavity. These vascular papules or polyps have a diameter of up to 1.5 cm, and ulcerate and bleed due to minor trauma. Histologically, lesions are superficially located. The surface is commonly ulcerated with granulation tissue. The lesions are circumscribed capillary proliferations, with a lobulated architecture, often adjacent to larger vessels. The endothelium shows slightly enlarged, plump nuclei without atypia, and mitotic figures can be seen, and may be numerous. Endothelial cells and a continuous outer layer pericytes are immunohistochemically highlighted by vascular markers (CD31, CD34, ERG) and SMA, respectively. The lobules are surrounded by thick bands of fibrous tissue. Neutrophils are often present. Pedunculated lesions can be ligated, while other forms are best treated surgically [20, 26, 31].

## Epithelioid Hemangioma (EH) (Synonym: Angiolymphoid Hyperplasia with Eosinophilia)

**Fig. 6:**
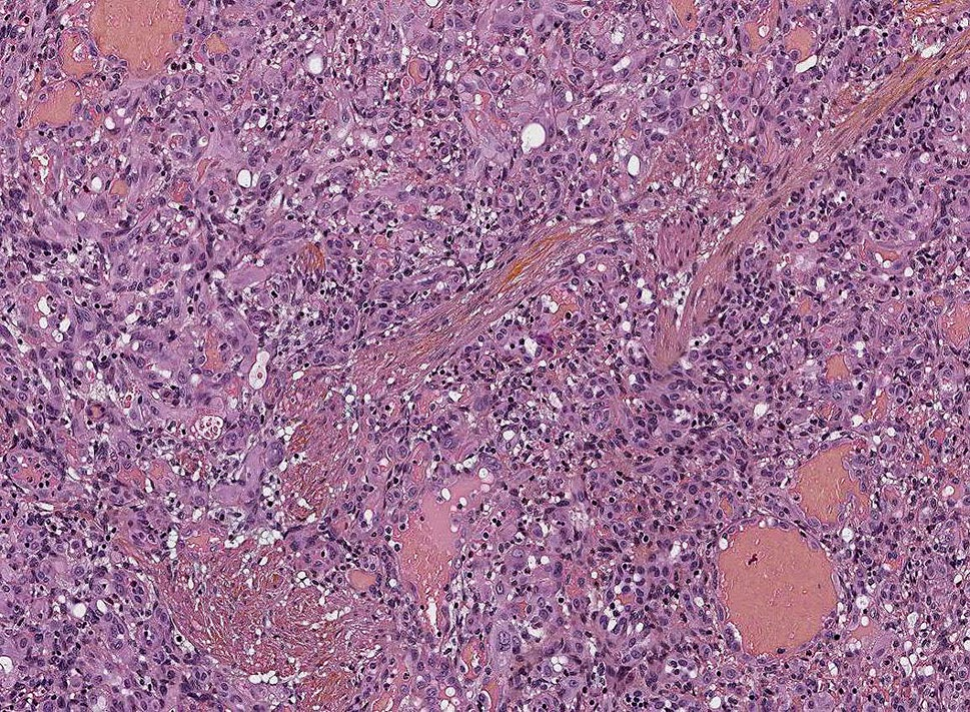
Epithelioid hemangioma. Note compact and vasoformative areas of epithelioid endothelial cells. The stroma indicates a lobulated architecture

EH is an uncommon benign vascular tumor characterized by epithelioid morphology. The head and neck region is a common site. EH originates in skin, subcutis, soft tissue and bone and may occur multifocally (in up to 50% of cases). There are three histologic subtypes: conventional, cellular and angiolymphoid hyperplasia with eosinophilia. EH is distinguished by a lobular architecture and a zonated pattern with tightly packed epithelioid cells located centrally, and maturating towards the periphery, which shows well-formed vessels. Intracytoplasmic vacuoles are often present. There is typically mild cytologic atypia, with mitotic figures (Fig. 6). A fibromyxoid stromal reaction is often present, and vascular invasion may be seen. The latter, as well as multifocality and solid central sheets of tumor cells can simulate a more aggressive lesion, especially when there is involvement of lymph nodes. However, muscle specific actin and SMA highlight the pericytic cuff, arguing against epithelioid hemangioendothelioma (EHE) and angiosarcoma (AS). Furthermore, the typical strands and cords of tumor cells and myxohyaline stroma of EHE are not present in EH. An inflammatory reaction with eosinophils is variably present. Kimura disease, a systemic disease with prominent inflammation, lymph node involvement and peripheral eosinophilia is a morphological differential diagnosis but is clinically distinct [33–38]. Nuclear FOSB reactivity is reported in a variable subset of cases, including all angiolymphoid hyperplasia with eosinophilia cases [35, 37, 38].

Genetically, EH harbor *FOS* and *FOSB* rearrangements, although these findings are very rare in the superficially located head and neck cases and analyses are not very helpful in this respect [35, 37, 38].

Spontaneous regression has been documented, however, circa 30% of cases recur locally. The optimal treatment is complete excision [37].

## Epithelioid Angiomatous Nodule (EAN)

EAN is a benign cutaneous lesion in the spectrum of epithelioid vascular proliferations. Middle-aged adults are mainly affected with an age range from 15 to 79 years. Lesions are rarely described in the head and neck region, with most frequent occurrence in the face and nasal mucosa. Histologically, these are well-circumscribed nodular neoplasms typically present intradermally or intramucosally, although extension into subcutis/submucosa is possible. There is a sheet-like proliferation of epithelioid cells containing abundant eosinophilic cytoplasm. Vascular channels are always present and may be very subtle with the presence of intracytoplasmic lumina. The nuclei display vesicular chromatin and prominent nucleoli. Vessels adjacent to the lesion are dilated. There is hemosiderin deposition and an inflammatory reaction. Endothelial markers are expressed immunohistochemically with SMA highlighting the pericytic layer of the vascular channels. EAN may easily be mistaken for a malignant lesion (epithelioid hemangioendothelioma or epithelioid angiosarcoma). However EAN is well-circumscribed, and shows no cytologic atypia in contrast to these malignant neoplasms, particularly angiosarcoma [37, 39]. CAMTA1 immunohistochemistry or detection of the typical fusion genes of EHE can also help to discriminate EAN from EHE [40]. Negativity for FOSB suggest possibly a distinct pathogenesis from epithelioid hemangioma [36]. Simple excision is the treatment of choice [37, 39].

## Vascular Malformations

Vascular malformations may contain venous, capillary, lymphatic or arterial components in any combination and are associated with overgrowth syndromes [26, 41]. Due to mosaic somatic mutations that arise during embryogenesis, or more rarely germ-line mutations, developmental errors occur in the vascular system. These lesions grow slowly and proportionately with the overall growth of the child, and persist throughout life. Histopathologic distinction from hemangioma can be difficult without patient history, and reactive changes such as vascular ectasia, recruitment of collateral vessels, organizing thombi, hormonal or angiogenic modulation, neovascularization in response to abnormal hemodynamics and signs of regression can blur the typical features. Additionally, differing terminology can cause confusion [26]. Of note, the endothelium of vascular malformations does not express GLUT1, which is characteristic for infantile hemangioma [26, 42]. WT1 immunohistochemistry can be helpful in discriminating vascular neoplasms from malformations with absence of cytoplasmic staining in lymphatic, venous and capillary malformations [42]. Mutationally affected genes include *PIK3CA, TEK (TIE2), AKT, GNAQ, GNA11, KRAS, NRAS, PTEN* and *RASA* [43].

## Cutaneous Capillary/Venulocapillary Malformations (Port-Wine Stains)

These lesions arise in the skin, and can be focal or multifocal, consisting of mature capillaries and venules with sometimes extension into subcutis. The vessels are rounded, and lined by elongated endothelial cells and pericytes. There is no mitotic activity. The vessel walls become fibrous and loosely organized smooth muscle fibers are present [26].

## Venous Malformation (VM)

VMs are characterized by abnormal collections of veins, that are superficial or deep, diffuse or localized, and solitary or multiple. Veins show a variable diameter and a variable thick smooth muscle layer, absence of an internal elastic membrane, and inconspicuous endothelium. Small venules or capillaries may also be present. Thrombi and phleboliths with signs of recanalization (Masson lesions) are relatively common. The latter can be confused with malignancy [26].

## Glomuvenous Malformation (GVM) (Synonym: Glomangioma)

This is a frequently multifocal neoplasm occurring in infants, children and adolescents. It accounts for 10% to 20% of all glomus cell lesions. GVMs are superficially located and often disseminated over a large cutaneous area showing multiple to confluent red-blue nodules. GVMs are less painful when compared with adult-type glomus-tumors. The expansion of the lesion makes surgery less amenable and subtotal resected lesions may progress. Laser and sclerotherapy may be helpful. Histologically it resembles a venous malformation with dilated veins surrounded by layers of glomus cells, and sometimes with organized thrombi [26].

GVMs are associated with mutations in the *GLMN* gene, which can be seen in both sporadic or hereditary cases [44].

## Arteriovenous Malformation (AVM)

AVMs are usually present at birth showing a significant arteriovenous shunting with raised skin temperature and pulsation. Hemorrhage and ischemia due to arterial steal are relatively common. Deep-seated lesions may become apparent at later age when shunting is low-grade. The histologic appearance of AVMs often varies, with arterioles, capillaries and venules present within a densely fibrous or fibromyxoid background, intermixed with numerous larger-caliber arteries and thick-walled veins. The arteries are often tortuous and the veins demonstrate intimal and adventitial fibrosis. Occasionally scattered dilated lymphatics may be present. The involved skin may show pseudoangiosarcomatous proliferation of small vessels probably due to arterio-venous shunt-related tissue ischemia [26].

## Lymphatic Malformation (LM) (Lymphangioma)

LMs originate commonly in skin and subcutis, and more rarely in soft tissue or viscera with localized/regional growth or diffuse involvement of many tissue planes or organ systems. Most LMs are present at birth or within the first 2 years of life, commonly when superficially located. They are often associated with significant overgrowth of soft tissue (and bone). Localization of LM in the tongue/oropharynx can cause airway obstruction. LMs can be divided into macro- and microcystic or combined lesions, depending on the cyst diameter with a cut-off of 0.5 cm. Whereas microcystic forms occur anywhere in the body, the macrocystic LMs most frequently arise in the neck, axilla, chest wall or groin. Macrocystic forms rarely regress spontaneously. Surgery, sclero- and lasertherapy are treatment options.

Microscopically, microcystic LMs consist of dilated small angular to rounded vessels, lined by flattened endothelium and rimmed by rare pericytes and little or no smooth muscle. The cysts are filled with clear fluid and small numbers of lymphocytes and in traumatized lesions with blood. LMs can include preexistent structures appearing free-floating. Macrocystic LMs possess valves, show thicker irregular coats of smooth muscle and/or fibrous tissue. Organized thrombi due to vessel wall injury or communication with the venous system may be present; this makes it difficult to distinguish veins from lymphatic structures suggesting a venous LM (see below). D2-40 staining of lymphatics helps to discriminate, while CD34 is mainly negative. Lymphoid aggregates are common [26].

## Venous-Lymphatic Malformation (VLM)

These low-flow malformations occur in Klippel–Trenaunay-Syndrome and sporadically. The histology variably combines features of VM and LM. When superficially located, an angiokeratoma-like lesion may be seen [26].

## Malignant Vascular Tumors

### Angiosarcoma (AS)

**Fig. 7:**
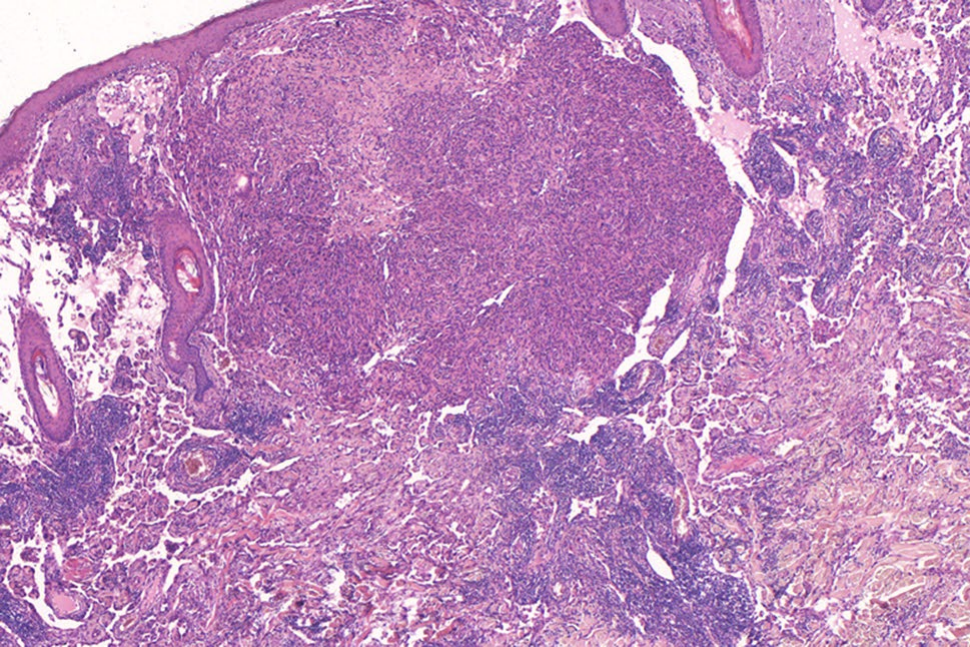
Low magnification showing a heterogeneous angiosarcoma consisting of an obvious vascular and a solid component

**Fig. 8:**
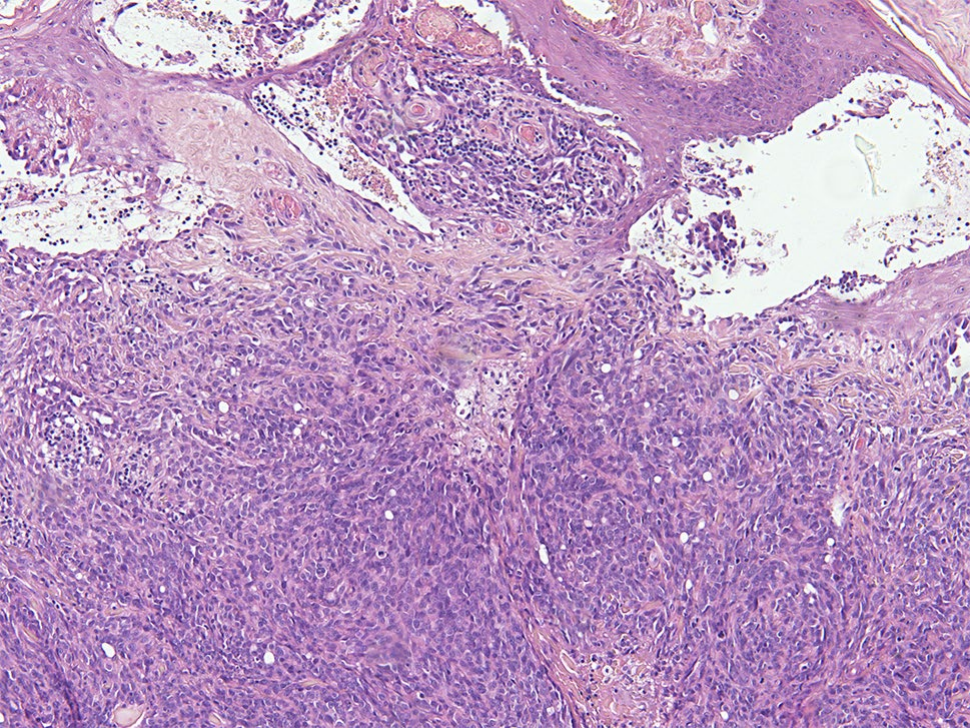
Angiosarcoma showing mainly solid sheets of atypical endothelial cells with rounded nuclei

**Fig. 9:**
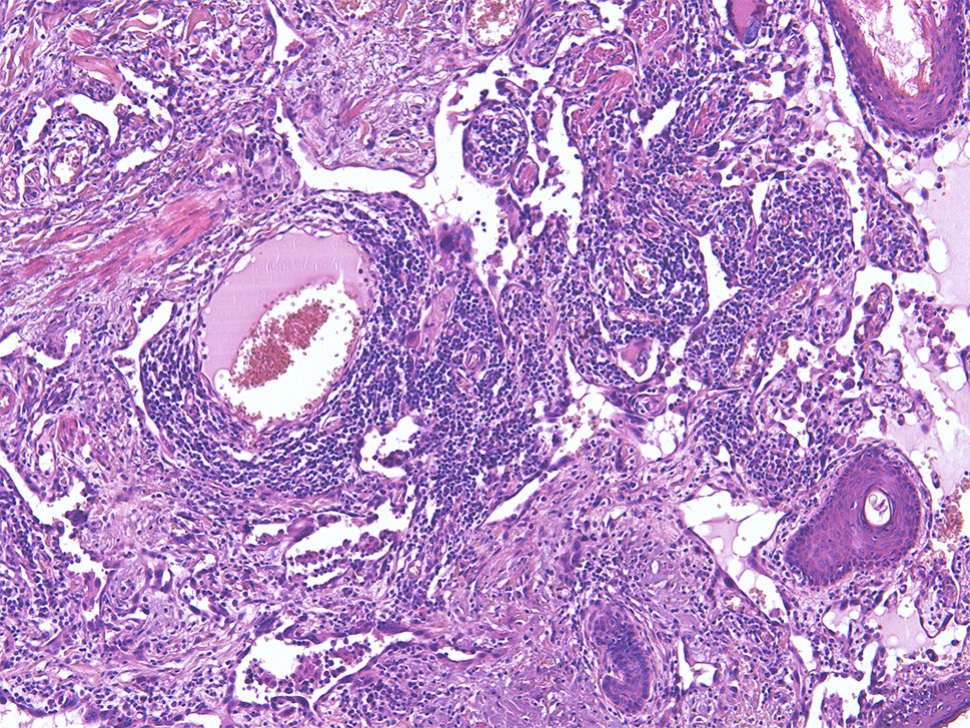
Angiosarcoma consisting of connecting atypical vascular channels lined by severely atypical endothelial cells. Note the inflammatory reaction

**Fig. 10:**
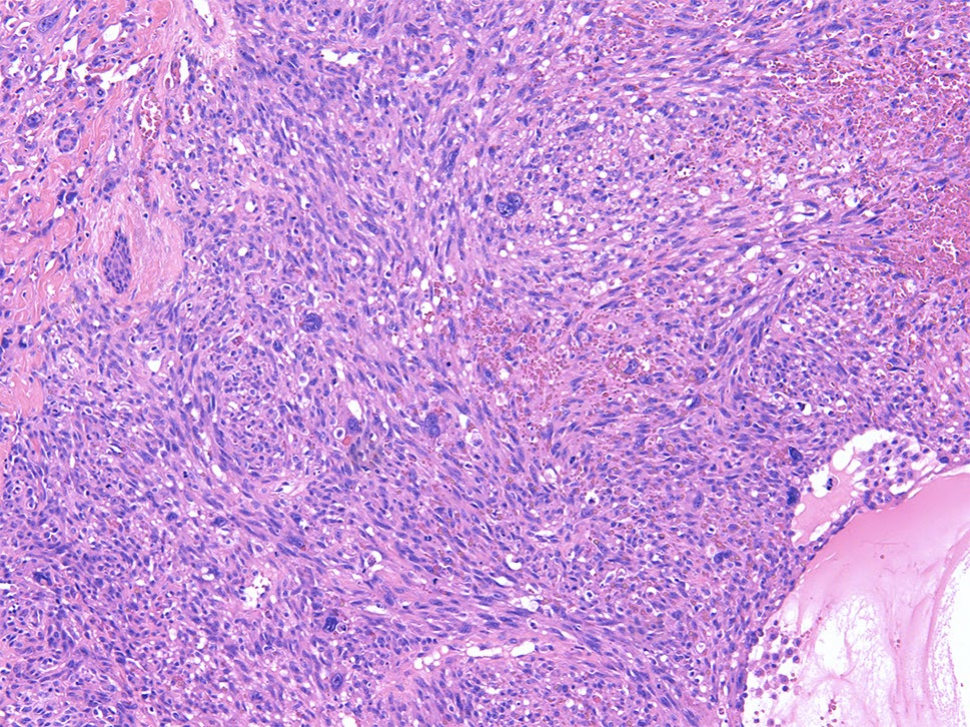
Angiosarcoma comprising pleomorphic tumor cells, which form subtle lumina

AS is a rare, aggressive sarcoma; one of its most common sites is the UV-exposed skin of the head and neck. Classically it occurs on the scalp of older Caucasian men mimicking a bruise or hemangioma. As tumors progress, lesions can become nodular, fungated and ulcerated. Local recurrence rates are high because of multifocality. Survival rates are generally poor, with a 5-year overall and disease specific survival of 26.5% and 48.3%, respectively. The traditional mainstay of AS treatment focuses on wide local excision with adjuvant radiotherapy [45, 46]. Chemotherapy produces partial response in the metastatic setting. Inhibitors of VEGF and VEGFR and broad-spectrum tyrosine kinase inhibitors have been reported to show clinical response with short-term outcomes. Propranolol and immunotherapy may be promising [45, 47]. The histologic spectrum of AS ranges from well to poorly differentiated, and features can vary within a single neoplasm (Figs. 7, 8, 9, 10).

The anastomosing vascular channels are ill-defined and diffusely dissect the dermal collagen when arising in the skin. A sieve-like architecture is a common feature. The atypical endothelial cells are usually multilayered, with enlarged, hyperchromatic nuclei and often prominent nucleoli. High-grade areas contain solid nests or sheets of tumor cells with spindle cell or epithelioid morphology with occasional intracytoplasmic lumina. Blood-filled spaces, high mitotic rate and necrosis are more often seen in less-differentiated tumors [48]. The most reliable immunohistochemical markers are CD31 and ERG with use in combination, while CD34 is variably present. Expression of keratins, partially observed in epithelioid AS should not be confused with carcinomas [48–50]. Of note, CD31 and ERG (own observation) can exceptionally be positive in atypical fibroxanthoma, which could be a diagnostic pitfall [51]. Genetically, various abnormalities have been described, including complex karyotypes, amplifications, mutations and fusion genes underpinning that AS constitutes a heterogeneous group of neoplasms [38, 52]. *MYC* amplification with expression of the corresponding protein is predominantly seen in cases secondary to radiation or UV-damage in contrast to primary angiosarcomas being rarely *MYC* amplified [38, 52, 53].

## Epithelioid Hemangioendothelioma (EHE)

EHE is a translocation-associated malignant endothelial neoplasm with a wide age distribution, although children are rarely affected. It may arise at any anatomic site including the head and neck region. Bone may be involved primarily or secondarily. There is a propensity for lymph node metastases and very rarely, a lymph node can be the site of origin [34, 54–56].

**Fig. 11:**
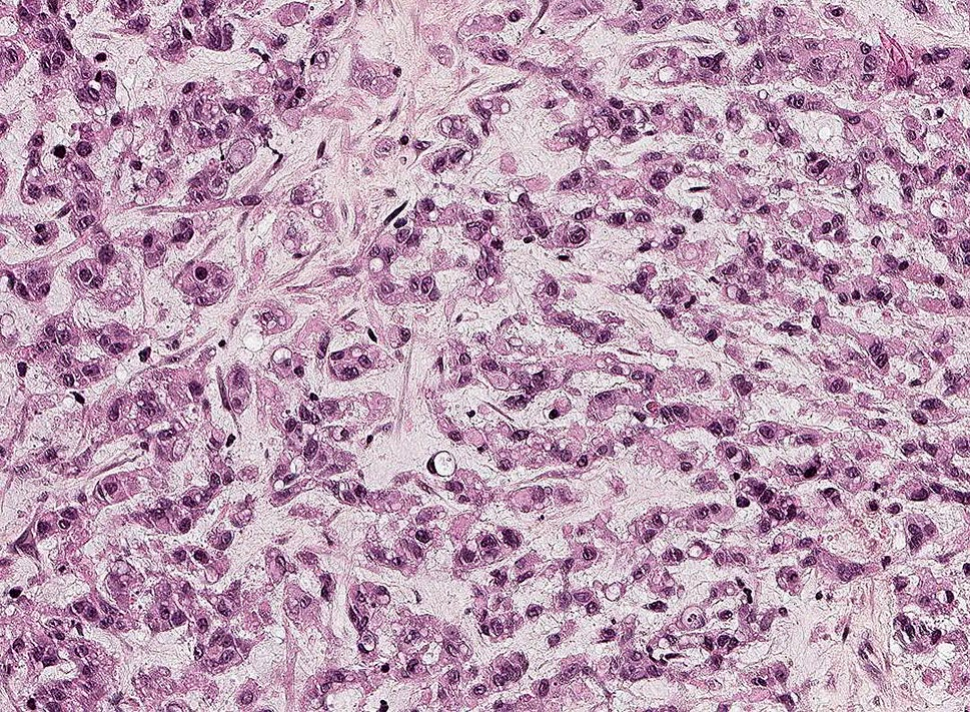
Epithelioid hemangioendothelioma consists typically of monomorphic epithelioid cells arranged in cords and nests. The stroma is variably fibromyxoid

Macroscopically, tumors are nodular or multinodular, with a pale solid cut surface with variable, commonly subtle hemorrhage. Histologically, lesions are infiltrative, consisting of epithelioid, histiocytoid and/or spindled cells arranged in strands and nests within a myxohyaline or fibrous matrix. Nuclei typically show little atypia. Marked nuclear atypia, observed in approximately one-third of cases, can be confused with epithelioid angiosarcoma. However, mitotic figures in EHE are typically scarce in comparison to angiosarcoma. There is a typical hyaline cytoplasm wherein sporadic vacuoles representing vascular lumina (so-called blister cells) (Fig. 11). More obvious vasoformative structures are present in a small subset of cases. The most reliable vascular markers for EHE are CD31 and ERG. Variable expression of keratins is seen in approximately 30% of cases, and may lead to confusion with carcinomas, myoepithelial tumors or mesothelioma, however expression of vascular markers is absent in these neoplasms [54, 57, 58]. *WWTR1*-*CAMTA1* is the most common fusion gene, while *YAP1*-*TFE3* is present in a small subset of cases [34, 55, 56]. By immunohistochemistry, tumors with these gene fusions are nuclear positive for CAMTA1 and TFE3, respectively [40, 56]. In contrast to epithelioid hemangioma, EHEs show no maturation towards the periphery and no SMA- or muscle specific actin-positive pericytic layer [33]. Furthermore, FOSB is not expressed [36].

Clinical behavior and prognosis depend on the primary site. Surgery is the treatment of choice in local disease. There is a risk of metastatic disease (synchronic or metachronic) and a significant mortality rate (up to 45%) [58–60].

## Vascular Tumors of Intermediate Malignancy

### Pseudomyogenic Hemangioendothelioma (PHE) (Synonym: Epithelioid Sarcoma-Like Hemangioendothelioma)

This distinct vascular tumor rarely occurs in the head and neck region. It mainly develops in young adults with male predominance. Lesions originate superficially and/or deeply with multifocal/multicentric disease in two-thirds of patients, with possible involvement of multiple tissue planes. The lesions recur locally after surgical treatment. Nodal and distant metastases have been rarely reported [50, 61, 62].

**Fig. 12:**
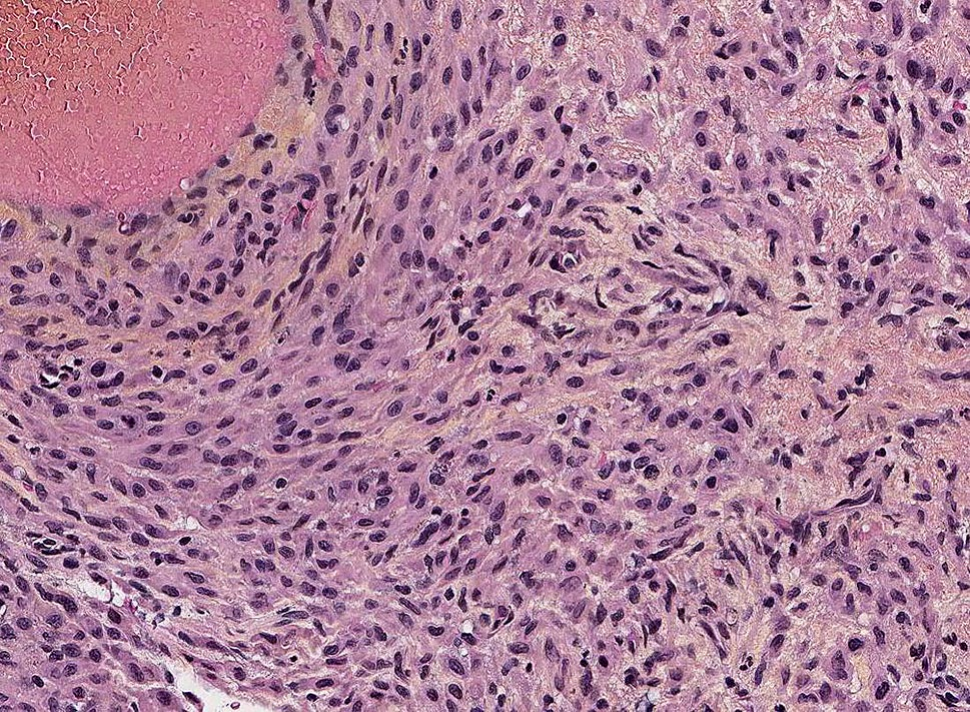
Pseudomyogenic hemangioendothelioma consisting of plump myoid cells; obvious vascular differentiation is absent

Macroscopically, tumor nodules are firm, grey-white and ill-defined. Microscopically, neoplasms show infiltrative nodules comprising sheets and fascicles of plump spindle cells with a myoid appearance and/or epithelioid cells with epithelioid sarcoma-like appearance. The cells are plump showing mild nuclear atypia, and eosinophilic cytoplasm. Pleomorphism and severe atypia are rarely present. There is typically mild mitotic activity. Obvious vascular differentiation or prominent hemorrhage are absent (Fig. 12). In some lesions vascular invasion is seen. Often, an inflammatory reaction is present showing mainly neutrophils. Necrosis is rarely detected [50, 61, 62].

Immunohistochemistry shows variable positivity of CD31 and strong expression of ERG. In contrast to epithelioid sarcoma, which also may express ERG, EMA and CD34 are negative. INI1 absent in epithelioid sarcoma is retained in PHE. Keratin AE1/3 is diffusely positive while other keratin-cocktails are negative [50, 61, 62]. Strong and diffuse nuclear staining of FOSB is due to *FOSB* rearrangement [36]. *SERPINE1* and *ACTB* are the known fusion partners [38, 63–65]. Rhabdomyoblastic/myoid tumors and inflammatory myofibroblastic tumors do not express vascular markers [25].

## Kaposiform Hemangioendothelioma (KHE)/Tufted Hemangioma (TA)

**Fig. 13:**
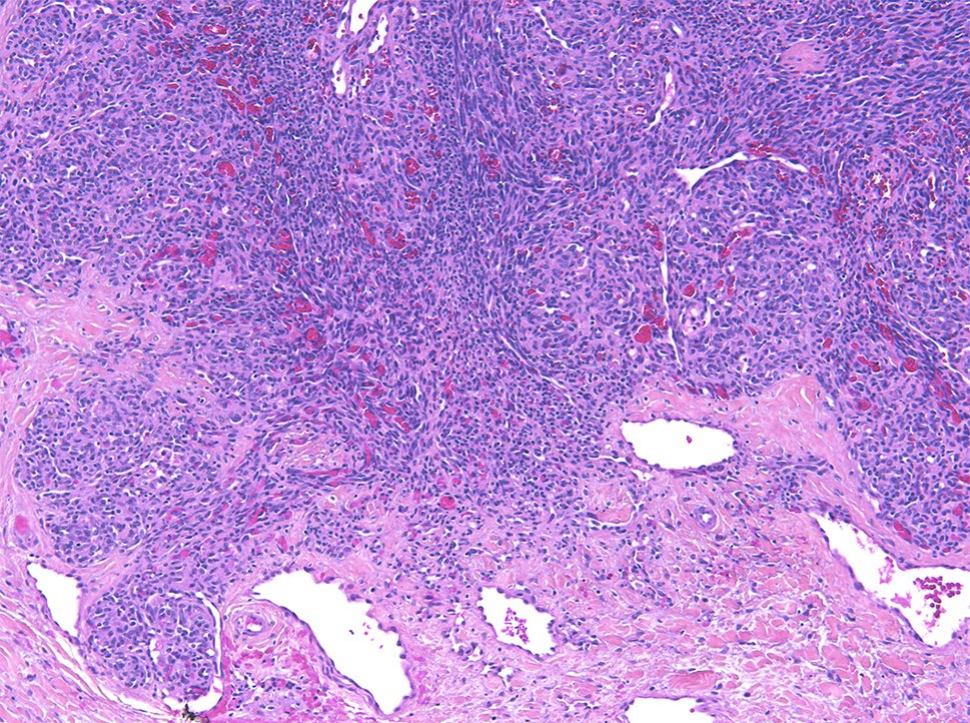
Kaposiform hemangioendothelioma with a lobulated architecture and a subtle slit-like vasculature formed by monomorphic spindled endothelial cells. Note the glomerular structures with more rounded tumor cells

KHE is a rare, locally invasive vascular neoplasm occurring mainly in infancy and childhood with an estimated incidence of less than 1 per 100.000 [66]. When arising in the head and neck area, the most frequent sites are the neck, followed by face and scalp, tympanomastoid region, oropharynx and paranasal sinuses. Patients present with a rapidly enlarging mass located superficially and/or deeply. Possible other symptoms are respiratory distress, cranial nerve palsy, epistaxis, hemoptysis, otorrhagia and hyposmia [67]. Kasabach–Merritt-phenomenon or disseminated intravascular coagulation is an adverse clinical condition often associated with a deep anatomic site [68–70]. Tufted hemangioma belongs to the spectrum at the benign end [27, 71]. Macroscopically, KHEs are ill-defined, firm and/or spongy and hemorrhagic. Histologically, lesions consist of spindled endothelial cells forming slit-like lumina arranged in nodules and sheets with infiltrative growth. Glomeruloid structures and larger lymphatic channels are scattered throughout (Fig. 13) [68].

TAs are composed of lobules of capillaries arranged in a cannonball pattern within the dermis and possibly subcutis variably surrounded by a desmoplastic stroma. The capillaries are tightly packed, similar to the arrangement seen in KHE. Peripherally there are larger thin-walled vessels. Endothelial cells are spindled, as in KHE. Microthrombi may be present [27].

Vascular markers (CD31, CD34 and ERG) are positive in both lesions, and D2-40 and PROX1 are also expressed [27].

Reported genetic changes are scarce with *GNA14* mutations described in one case each of KHE and TA [72]. However, *GNA14* mutations are also reported in congenital hemangioma, hepatic small vessel neoplasm and anastomosing hemangioma which are potential differential diagnoses [72–74]. The architecture of TH and KHE are unique and hemangiomas have a pericytic layer around the endothelial cells. Whether kaposiform lymphangiomatosis is part of KHE needs further clarification [75]. Spindle cell hemangioma (SCH) shows a vague resemblance to KHE but the prominent cavernous component of SCH is absent in KHE. Furthermore SCH is typically located on the extremities and harbors and *IDH1/2* mutation [68, 69, 76].

Treatment with sirolimus has been shown to be successful. Surgery of KHE is often unattainable due to extent of the lesion. Single and combination chemotherapy have been used to treat KHE patients. Local excision of TAs is adequate [69].
